# Patients’ and healthcare professionals’ views on a specialist smoking cessation service delivered in a United Kingdom hospital: a qualitative study

**DOI:** 10.1186/1617-9625-12-2

**Published:** 2014-01-29

**Authors:** Manpreet Bains, John Britton, John Marsh, Leah Jayes, Rachael L Murray

**Affiliations:** 1 UK Centre for Tobacco and Alcohol Studies and Division of Epidemiology and Public Health, University of Nottingham, Nottingham NG5 1 PB, UK

**Keywords:** Smoking cessation, Support, Secondary care, Provider-patient relations, Qualitative

## Abstract

**Background:**

Hospital admission provides a powerful opportunity to promote smoking cessation. We explored patients’ and healthcare professionals’ (HCP) views of a specialist smoking cessation service comprising systematic smoking ascertainment, default provision of pharmacotherapy and behavioural counselling at the bedside, and post-discharge follow-up, in a clinical trial in a United Kingdom teaching hospital.

**Methods:**

Semi-structured interviews with 30 patients who were offered the intervention, and 27 HCPs working on intervention wards, were audio-recorded, transcribed verbatim and analysed using thematic analysis.

**Results:**

The shock of being admitted, and awareness that smoking may have contributed to the need for hospital admission, caused many patients to reassess their quit intentions. Most patients felt the service was too good an opportunity to pass up, because having long-term support and progress monitored was more likely to result in abstinence than trying alone. Had they not been approached, many patients reported that they would have attempted to quit alone, though some would have been discouraged from doing so by pharmacotherapy costs. Service delivery by a specialist advisor was favoured by patients and HCPs, largely because HCPs lacked time and expertise to intervene. HCPs reported that in usual practice, discussions about smoking were usually limited to ascertainment of smoking status. Timing of service delivery and improved co-ordination between service staff and inpatient ward staff were matters to address.

**Conclusions:**

A hospital-based specialist smoking cessation service designed to identify smokers and initiate cessation support at the bedside was deemed appropriate by patients and HCPs.

**Trial registration:**

Trial registration: ISRCTN25441641.

## Background

Due to its highly addictive nature, smoking remains widely prevalent in high and low income countries [1,2]. Smoking causes significant harm to health, particularly through coronary heart disease, stroke, respiratory diseases and various cancers, and reduces average life expectancy by 10 years relative to never smokers [3,4]. Smoking cessation interventions are effective and highly cost-effective [5,6], and delivery of smoking cessation support is widely recommended in many healthcare provision guidelines [7-9].

Many people admitted to hospital are smokers. Smoking accounts for nearly half a million hospital admissions each year in the United Kingdom (UK) [10], and the coincidence of an episode of illness with the enforced abstinence from smoking that admission typically requires makes hospital admission a particularly important opportunity to promote smoking cessation. Hospital-based smoking cessation interventions providing behavioural counselling, pharmacotherapy and follow-up for at least one month after discharge, are highly effective [11]. However, despite guidelines recommending that hospital care should include such interventions [7,9,12], they have not to date been widely incorporated into routine clinical practice [13-15]. A recent survey found that less than a quarter of 121 countries surveyed reported routine recording of patients’ smoking status in medical notes [16], suggesting that opportunities to provide even brief advice are being missed.

It is therefore important to optimise the reach and delivery of hospital smoking cessation services, and to ensure that the views of service-users and healthcare professionals (HCPs) are given proper consideration in their development [17]. However, qualitative research in these groups is scarce, and limited primarily to community-based settings [18,19]. We have recently reported a cluster-randomised controlled trial demonstrating that systematic integration of a hospital-based specialist service substantially increased uptake and delivery of smoking cessation interventions in acute medical wards in a UK hospital [20]. We now report the results of qualitative interviews conducted with patients and HCPs to explore their views of the service, and how it might be improved.

## Methods

### Study design and sample

Eighteen wards in a UK teaching hospital were allocated, in a cluster-randomised design, either to deliver systematic smoking cessation interventions to all admitted patients who smoked, or to deliver usual care. According to the study protocol, researchers approached patients identified as smokers on intervention wards to establish desire to quit. Patients who accepted the offer of support, hereafter referred to as service-users, were visited at the bedside by a specialist smoking cessation advisor, typically on the same day, who provided one-to-one behavioural support and pharmacotherapy, and appropriate follow-up support. Irrespective of whether patients wanted to quit or accepted the offer of support, all were asked to consent to be contacted by the research team at four-weeks and six-months following discharge to ascertain smoking status and use of smoking cessation support.

To explore the appropriateness of the service comprising the intervention, smokers who were contacted between May and August 2011 as part of follow-up were invited to take part in an interview, and comprised both those who accepted (service-users) and did not accept (non service-users) the support. Those expressing an interest were posted an information sheet and consent form, and were contacted by the interviewer (M.B.) a few days later to ascertain whether they wished to take part. For those giving verbal consent, a date for interview was arranged, either via telephone or at the patient’s home. Written consent was obtained from each participant before the interview commenced (via post for telephone interviews). HCPs working on the nine wards delivering the intervention were also invited to take part in an interview, and those expressing an interest were given an information sheet and contacted at least 24-hours later to ascertain whether they wished to participate. Those giving written informed consent were interviewed in a quiet room in the workplace. Participants were informed that transcripts would be anonymised, treated confidentially and that they were free to withdraw at any point during the interview, if they so wished. All interviews were digitally audio-recorded. On average, patient interviews lasted 25 minutes and HCPs’ lasted 15 minutes. The study was approved by Nottingham Research Ethics Committee (10/H0403/30).

### Interviews

Two semi-structured interview guides were developed: one for patients and another for HCPs. The interview guide for patients explored both service and non service-users’ views on the smoking cessation service they were offered, recollections of the initial approach, reasons for acceptance or non-acceptance of support, the nature of support received before and after discharge, and quit intentions before admission. Interviews with HCPs explored views on the importance of quitting for patients admitted to their respective wards, levels of usual care provision of smoking cessation advice, and views about the specialist service.

### Data analysis

An external specialist transcription company transcribed the interviews, verbatim. The interviewer removed any identifiers and quality checked each transcript. Participants were assigned a unique code; for patients this included the ward that they were admitted to, their gender and age, and for HCPs this was the specialty they represented. Thematic analysis was applied to the data [21], where three researchers (M.B., J.M. and L.J.) analysed the transcripts independently, noting emergent themes and sub-themes. The initial coding hierarchies derived by each researcher comprised similar themes. The hierarchies were discussed between the researchers and resulted in a final codebook of three core themes: relation between smoking, the hospital admission and provision of advice, service delivery which had corresponding sub-themes (appropriateness, timing and recollection of approach, reasons for acceptance and exploring whose role it is to deliver support), and service-users’ views of the support received post-discharge. Following this, NVivo 9.2 (QSR International Ltd, Melbourne, Australia) was used to manage extracts from the transcripts according to the themes that they represented and which reflected the overall views of the participants.

## Results

### Participant characteristics

Between May and August 2011, 174 patients, of whom 116 (67%) were service-users, were contacted for follow-up and invited to take part in the interview study. Of these 174, 44 (25%) expressed an interest and were sent the information sheet and consent form, and 30 (22 service-users and 8 non service-users) were interviewed. When comparing those who were interviewed with the 144 that were not, mean age and distribution of gender were similar (55 years and 54 years, male = 67% and 65%), however the proportion of individuals smoking at four-weeks differed (30% and 49%). Non service-users’ reasons for non-acceptance were not wanting to quit, or feeling in control and able to quit without support.

Patients interviewed had been discharged from five specialty wards: cardiology (n =19), oncology (5), stroke (3), infectious diseases (2) and endocrinology (1). HCPs were recruited from a purposive sample of 35 staff representing all of these specialty areas, and 27 (77%) participated, from specialties comprising oncology (10), cardiology (6), stroke (4), renal (4) and infectious diseases (3). The sample comprised six charge nurses (22%), five deputy ward sisters/charge nurses (18%), three senior nurses (11%), five staff nurses (18%), two junior doctors (7%), one cardiac-rehabilitation nurse (4%), one stroke rehabilitation nurse (4%) and five discharge co-ordinators (18%). Most were female (18, 67%).

### Relation between smoking, hospital admission and provision of advice

Quitting had been on most patients’ minds for some time before their hospital admission (see the **'Relation between smoking and hospital admission and provision of advice’** subsection). Most had not planned how to go about quitting, but those that had stated that they would have attempted to quit without support. The shock of being admitted to hospital coupled with the fear of further ill-health resulted in most patients reassessing their quit intentions, particularly those who felt that smoking had contributed to their hospital admission. Some patients felt however that their admission was likely to be due to a number of lifestyle or hereditary factors rather than smoking alone; in some cases, conversations with HCPs appeared to strengthen these beliefs further. HCPs reported that in usual practice, after ascertainment of smoking status few would probe patients’ intentions to quit, and discussions with patients varied and depended on the nature of a patient’s condition or disease. Some HCPs admitted that they often refrained from initiating a discussion about smoking with patients diagnosed with advanced disease, such as cancer or renal failure, out of concern that it may induce levels of undue stress that may outweigh the benefits of quitting, even though some were seemingly aware of benefits of quitting even in terminal palliative care.

### Relation between smoking and hospital admission and provision of advice

*Then this frightened me and when they took us to hospital I thought 'I've got to stop now, I can't do it anymore'. That was it, I haven't had one since.***Endocrine, female, 66 yrs.**

*No, I’ve been told not, the cancer I had was not caused by smoking.***Oncology, female, 44 yrs.**

*The nurses and all that, they said it don’t help but it’s not the be all and say all of the problems they go on about with smoking, you've got folks dying of lung cancer that’s never smoked in their life so what does that prove? It [smoking] didn't help, it’s lifestyle as well.***Cardiac, male, 67 yrs.**

*I'd say that the patients who we tend to discuss it mostly with are the people who have had new transplants, new kidney transplants, at the point where we're discharging them home we're very much looking at their ongoing future health and promoting their health and maintaining the functioning of their new kidney. So when we do have patients who are smoking going home with a new kidney we will be very keen to discuss that with them.***Renal, Healthcare Professional.**

*It probably must have been two years when I first heard of [the local smoking cessation service], and, really, it’s been something that’s been, as I see it, it’s been promoted quite softly, really. It’s been like an ancillary service that’s there, sort of if you want to access it, and I haven’t really sort of actively promoted it on the ward, because I don’t feel like our group of patients, I don’t feel, really, that that’s appropriate, but I am aware that we’ve got their cards available on the ward to give out to the patients.***Oncology, Healthcare Professional.**

### Service delivery

#### Appropriateness, timing and recollection of the approach

The majority of patients stated that being approached by the research team to discuss their smoking behaviour and quit intentions was entirely appropriate, and was welcomed by most; particularly by those who were unaware of the existence of smoking cessation services (see the **'Appropriateness, timing and recollection of the approach’** subsection). Furthermore, most HCPs felt that the specialist service was the most suitable approach to help patients who wished to quit, and that patients were more likely to be receptive when being approached by individuals with the relevant skillset and adequate amount of time to discuss the subject and provide support promptly. It was clear that usual care hospital smoking cessation support practices were considered insufficient and ineffective, as reflected by reporting by HCPs that provision of NRT following admission was either scarce or delayed, and provided to encourage temporary abstinence rather than to support or promote a quit attempt. A small minority of patients and HCPs felt that discussing smoking at the point of admission was inappropriate, mainly because they were either still coming to terms with their reason for being admitted or they had just undergone a procedure which had left them feeling unwell. Such patients suggested that it would have been better if they had been approached closer to or soon after discharge. Consideration of the timing of service delivery was also highlighted by a number of HCPs, especially those from oncology, where it was suggested that delivery may need to be tailored according to specialty and where a patient was in their care pathway.

### Appropriateness, timing and recollection of the approach

*In some ways it seemed quite natural because you were in an environment where in theory smoking was heavily discouraged, it does say on both campuses that when you're inside the building or outside that they have a non-smoking policy and it was certainly around on the wards and there were certainly [local smoking cessation service] signs around the various hospitals, that it seemed a very 'natural’ environment to consider giving up smoking.***Stroke, male, 58 yrs.**

*I think it’s good that we’ve got the research team coming round and doing stuff like that and it takes a lot of the pressure off the nursing staff, because they’re seen as experts in smoking cessation, that helps a lot because we can give out certain information and we can guide them to [local smoking cessation service] and we can do our bit with regards to patches, but I think if you don't have the expertise in giving out the information then it’s difficult for patients and people that are in hospital to take you that seriously, if you've got somebody that comes round face to face, patients are more receptive to that than giving them a number to phone because they’ll probably look at the leaflet and think “I’ll do that another day”…***Oncology, Healthcare Professional.**

*…I think we weren’t probably as into the mindset of encouraging people to stop smoking, or support them, more like get them through this period thing, if that makes sense. I think sometimes patches may well have been prescribed more of the, let’s take the nicotine craving away for right now, rather than more of a long term plan if you like.***Stroke, Healthcare Professional.**

*I think it was two ladies that came round and asked me would I be prepared to go and sign up for a no smoking thing, I said “yeah, anything”, basically I wanted to get rid of them… Put yourself in the state of a patient who’s had a heart attack in the middle of the night, who’s been told that if he don't do this, he’s going to die, you've got wires coming out all over you, you're still partially dazed, whatever and the last thing you want is people standing by your bed saying, “do you want to do this, do you want to do that?”. Patients don't want that in the first couple of days, yes fine to do it but bloody hell, give the bloke time to either survive or die, in two days it’s too soon. I think the set up you've got from what I’ve seen of it is adequate, it’s purely the timing.***Cardiac, male, 72 yrs.**

*I think, as I said, mostly people when they're here they're having some sort of crisis in their life and I think that's the worst time for them to try and give up smoking… Yeah. It isn't the right time for them. I don't know whether you're aware that a lot of our patients who come here who do smoke they still smoke within the hospital grounds. We're supposed to enforce a no smoking hospital but it seems very unfair and unrealistic to enforce that on people who are very ill and are making an informed decision to carry on smoking.***Renal, Healthcare Professional.**

*I suppose it depends on how the patient is at the time of admission, because obviously some patients are quite poorly when they first come in, so it may be then that you leave it for, I don’t know, two or three days and then talk to them about it then.***Oncology, Health Professional.**

#### Exploring whose role it is to deliver support: specialist service versus HCPs

It was widely acknowledged by both patients and HCPs that the delivery of smoking cessation support in hospital settings was both warranted and important, and most felt that such a service should be provided by smoking cessation specialists rather than HCPs (see the '**Exploring whose role it is to deliver support: specialist service versus HCPs**’ subsection). Patients recognised that time constraints and lack of knowledge was likely to prevent HCPs providing comprehensive support; HCPs commonly reported these factors as barriers preventing them from addressing the matter in more depth. Consequently, most patients mentioned that discussions that they did have about smoking with their treating physicians or nurses failed to go beyond advising them to stop; hence, some patients felt that HCPs broached the subject in an inappropriate manner. HCPs admitted that they rarely went beyond ascertainment of smoking status, with some disclosing that they had seldom referred patients to a local smoking cessation service, describing the paper-based referral system to community services as inefficient and time consuming. Some HCPs did however recommend that raising the profile of the service amongst inpatient ward staff would have been beneficial, as HCPs’ knowledge and understanding of the process and nature of the study and service was mixed. Level of understanding seemed to have implications for the extent to which HCPs liaised with the research team to help identify patients that were smokers. Similarly, several patients recommended better co-ordination between the specialist service and physicians or nurses, where the latter could perhaps initiate the discussion about smoking cessation, and then refer on to a specialist service to provide further information and/or support.

### Exploring whose role it is to deliver support: specialist service versus HCPs

*I’m not sure that that’s necessarily a role that the doctors or nurses can handle along with all the other things that they’ve got to do if you see what I mean. I think it’s a subject, it’s an issue that it’s big enough in scale to warrant having specialists in every hospital really that are there to their deal with that particular issue.***Cardiac, male, 44 yrs.**

*I think nurses would be happy to do referrals. We often do referrals to diabetic teams, district nurse, tissue viability over notice. I don't think that would be a problem. Us giving more support I think would be the problem, we don't have enough time to sit with patients and give support as much as we'd like, should I say on dialysis, when patients first come for dialysis. So doing it again for smoking as well I don't think we'd have enough time to fully support our patients.***Renal, Healthcare Professional.**

*They talked to me, not at me, doctors talk at you, they talked to me, making me feel it was my decision, doctors want you to stop so they just nag.***Cardiac, female, 56 yrs.**

*I don't know any of the other nurses like myself would get involved in advising patients to stop smoking or to try and convince them to stop smoking, or to try and convince them to stop smoking or to have a lengthy chat about smoking, I’ve been here, I know most of my colleagues well and I don't think I’ve ever heard anybody on admission or in a general conversation, have a big discussion about smoking.***Oncology, Healthcare Professional.**

*From what I can see, the person in question comes along to the ward, has a look at the notes and see if the person wants to stop smoking or not. [Interviewer: was there any discussion about the study?] Not so much discussion as we were just told “don’t be alarmed if you see this person on the ward, they’re coming to do a study about smoking”, that was pretty much it, so then you know they’re there, that was it.***Oncology, Healthcare Professional.**

*I think that the doctors should suggest to the patient that there is help if you smoke, do you mind somebody coming round and having a chat with you, I think it should be the doctor who decides, well not decides, but asks the patient. Instead of people just coming round, because I feel that doctors know more about what’s up with the patient, and maybe it is, he’s never smoked, he hasn’t got a heart problem but he’s had a heart attack because of him smoking, that maybe people should be seen, I think it should be the doctor who advises you that you should be seen by these people.***Cardiac, male, 48 yrs.**

#### Reasons for acceptance

Other than wanting to quit because of their hospital admission, service-users accepted the support because they wanted to stop for their families (see the **'Reasons for acceptance’** subsection). Additionally, several participants disclosed that having long-term support and their progress monitored was more likely to result in achieving abstinence, rather than trying alone. Great appeal was associated with the way pharmacotherapy treatment and appropriate follow-up was arranged for service-users, meaning that little effort was required by them. Patients were asked what they would have done had they not been offered support, and the majority indicated that they would have attempted to quit alone, with only a few saying that they would have contacted a smoking cessation service. Moreover, some patients acknowledged that it was unlikely that they would have tried to quit due to the costs of NRT, which they believed made it cheaper to smoke.

### Reasons for acceptance

*To stop smoking, just I’d got to because my main reason was my daughter, my mother died when I was very young, I didn't want her losing her mum and my husband, I couldn't do it to him, I’d got to stop and them people helped me stop and I will always be grateful for that, the support and every day just like friends sat there nattering, is amazing, it really is amazing.***Cardiac, female, 56 yrs.**

*I still think the main thing was the fact that somebody else would do something about it, somebody would send me vouchers, someone would supply me with vouchers, somebody would ring me up once a week or once a fortnight or whatever it was.***Infectious diseases, male, 58 yrs.**

*I would have tried [if not approached], yes but it would have been the financial side of it… because I didn't know anything about [local smoking cessation service] or anything then, it would have been the financial side and it sounds stupid, it’s still cheaper to smoke than it is to buy the nicotine replacement products and what do you do?***Cardiac, female, 58 yrs.**

### Service-users’ views of the support received post-discharge

To ensure the support initiated during the inpatient stay was maintained, follow-up support was arranged and NRT was provided to service-users before discharge, where possible. Telephone support was favoured by most service-users because they did not feel well enough to attend a clinic-based setting, felt uncomfortable in group settings, or because this was more convenient than attending a clinic (see the '**Service-users’ views of the support received post-discharge’** subsection). These participants described that they received weekly phone calls to discuss their progress and that they were sent vouchers to replenish NRT or a prescription was arranged via their general practitioner and stated that this arrangement suited them well. In contrast, some service-users found attending a clinic setting on a weekly basis helpful, particularly for one who attended a group setting. Irrespective of the type of follow-up, most service-users believed it was important to speak with the same advisor because this helped to build a good relationship. Service-users underscored the importance of the support that was initially offered and subsequently arranged during their hospital stay as being of immense value, with most service-users indicating that they could not have quit without it, or would not have even tried to quit.

### Service-users’ views of the support received post-discharge

*…they came back and did another one [CO reading] and just to see me really before I was going home and just to say that everything was in place, it was all just easy. [Local smoking cessation service] contacted me when I got home and took over from there, my prescriptions and everything, I just had to go to the doctor and get them, I didn’t have to do anything.***Cardiac, female, 58 yrs.**

*It was the best option for me as well because obviously I weren’t 100%, I’m very busy with the children anyway, with my busy schedule, I might not have turned up [appointment at clinic], so having someone on the other end of the telephone was so much easier than having to physically get myself somewhere…No, no I don’t [think could have quit without support]. Because I’d have done my usual “I’ll just have this one”, “I’ll just have a couple today and I’ll pack up tomorrow”, “I’ll wait until next week and then…”. that would have been it, I wouldn’t have done it…It would have been harder without the support because I don’t think I would have then had the [nicotine replacement therapy] that I had and I’ve have been a bit more ratty!***Oncology, female, 44 yrs.**

## Discussion

The shock of being admitted, fear of further ill-health, and that most patients acknowledged that their hospital admission was likely to be attributed to smoking, resulted in reassessment of quit intentions, which supports the window of opportunity to promote and provide smoking cessation support at the bedside. Service delivery by specialist advisors was favoured by patients and HCPs, and thus such approaches may help to address barriers preventing HCPs providing optimal support at present. Our findings from patients who did not accept the offer of support highlight that non-acceptance was attributable to personal reasons such as not wanting to quit or wishing to try without support, rather than something attributable to the service itself; however it is possible that improvements could be made to the service to engage more of those who declined.

The value of having access to specialist smoking cessation support following hospital admission is supported by our findings that had they not been offered support, most of the service-users would have attempted to quit alone, and hence achieved lower quit rates than achievable with effective, evidence-based support; whilst others would not have even tried, citing the cost of pharmacotherapy as the primary reason. Similarly, the service exposed patients to local smoking cessation services that they were not aware of previously. Ultimately, patients felt that the service was too good an opportunity to pass by, because little effort was required on their parts, support and pharmacotherapy were provided or organised and follow-up support was arranged prior to discharge; the importance of the latter has been reported previously [22,23].

The main trial findings shows delivering cessation support via specialist services rather than reliance on HCPs is an effective approach; where support initiated at the bedside, followed up with community referral after discharge doubled CO validated four-week continuous quit rates in the intervention group, and significantly increased the uptake of behavioural support, use of pharmacotherapy and referral to and uptake of community support after discharge [20]. This complementary qualitative evaluation shows the approach was favoured by patients and HCPs, because the latter lacked time and expertise to address the matter effectively. This is in line with previous findings suggesting that some HCPs believe they do not have a role in advising patients to quit [24,25]; this along with barriers such as underestimation of smoking-related disease risk and misperceptions regarding the harmfulness of nicotine prevent HCPs providing support [26-32]. Although the HCPs we interviewed comprised mostly of nursing staff, most admitted that discussions about smoking generally failed to go beyond ascertainment of smoking status, or were dependent on their judgements of which patients would benefit most. This suggests lack of awareness of the sound evidence base highlighting quitting is beneficial for all patients, and that guidelines including those available in certain specialties such as oncology (e.g. for lung cancer) are not being adhered to [7,9,12,33-35], and this needs to be addressed. Hence, results from previous studies [14] showing limited smoking cessation support documented in patients’ notes may be reflective of actual practice, rather than under-recording by HCPs. The provision of pharmacotherapy by HCPs was also concerning, where we found that there was no systematic process in place, other than providing it to encourage temporary abstinence rather than to promote a quit attempt. However, HCPs’ preoccupation with informing patients that hospital grounds are smoke free may account for this somewhat.

The content of the service was rated highly by both patients and HCPs. These findings are in line with a recent study that also reported patients appreciated the opportunity to embark upon a quit attempt during their hospital stay, though data from patients declining the offer of support was not collected [36]. By collecting this data, we were able to reaffirm that non-acceptance was attributable to reasons such as not wanting to quit, rather than the service itself. However, both patients and HCPs identified that timing of delivery requires further consideration and may need adapting according to specialty or where patients were in their pathway. At times, non-service users found it difficult to recall the initial discussion they had with the researcher about what the service involved, usually because they were approached soon after an operation, health event or following chemotherapy, and in hindsight they wished they had accepted the offer of support. However, because interviews took place either four-weeks or six-months post-discharge, recall bias may also account for these findings. Finally, patients and HCPs felt that improved co-ordination between intervention staff and inpatient ward staff was necessary, and this was reflected by HCPs’ varied level of understanding of the service. For future interventions, it is recommended specialist cessation service providers engage with inpatient ward staff, because this could impact service delivery and its effectiveness.

### Strengths and limitations

As these results are based on one intervention in a single hospital, and include interviews with a small proportion of self-selecting patients comprised largely of service-users, it is not known how representative these patients’ views are of all those that were admitted and eligible to use the service. Similarly the sample of HCPs interviewed lacked diversity, involving mainly nurses. Hence, issues regarding self-selection bias particularly regarding patients must be noted, and thus may have resulted in more favourable accounts of the service. However, collecting data from patients who declined the offer of support indicates that non-acceptance did not seem to be attributable to the service. Not having the views of smokers who were approached but did not provide consent to be followed up is also a limitation, particularly because this group may have provided alternative views on the appropriateness of the service. Engaging such groups in research remains a challenge [37]. Despite these limitations, these findings provide an account of how such approaches are likely to be perceived by patients and HCPs in secondary care, generally.

## Conclusions

Initiating smoking cessation support during an inpatient stay via a specialist service was considered appropriate and was favoured by patients and HCPs, rather than reliance on HCPs. Such approaches would overcome barriers preventing HCPs from intervening and may help to ensure support is provided consistently. To ensure optimal service delivery is achieved, research regarding timing of delivery, particularly according to specialty is recommended.

## Competing interests

The authors declare that they have no competing interests.

## Authors’ contributions

All authors were involved in the conception of the project and development of the interview guides. MB carried out the interviews, analysed the data and drafted the manuscript. JM and LJ assisted with participant recruitment, analysis and interpretation of the data. JB, JM, LJ and RLM reviewed the manuscript. All authors read and approved the final manuscript.
